# Nano-cellulose based nano-coating biomaterial dataset using corn leaf biomass: An innovative biodegradable plant biomaterial

**DOI:** 10.1016/j.dib.2017.12.046

**Published:** 2018-01-05

**Authors:** A.B.M. Sharif Hossain, Musamma M. Uddin, Vajid N. Veettil, Mohammad Fawzi

**Affiliations:** a Program of Biotechnology, Department of Biology, Faculty of Science, University of Hail, Hail 2440, Saudi Arabia; b Biotechnology Program, Institute of Biological Science, Faculty of Science, University of Malaya, Kuala Lumpur, Malaysia

**Keywords:** Nanocellulose, Nanobioplastic, Nanocoating, Biodegradable, Corn leaf

## Abstract

The nanocellulose derived biodegradable plant biomaterial as nano-coating can be used in the medical, biomedical cosmetics, and bioengineering products. Bio-plastic and some synthetic derived materials are edible and naturally biodegradable. The study was conducted to investigate edible nano-biopolymer based nano-coating of capsules and drugs or other definite biomedical materials from corn leaf biomass. Corn leaf biomass was used as an innovative sample to produce edible nano-coating bioplastic for drug and capsule coating and other industrial uses. The data show the negligible water 0.01% absorbed by bio-plastic nanocoating. Odor represented by burning test was under the completely standard based on ASTM. Moreover, data on color coating, tensile strength, pH, cellulose content have been shown under standard value of ASTM (American standard for testing and materials) standard. In addition to that data on the chemical element test like K+, ${\mathrm{CO}}_{3}^{--}$, Cl^-^_,_ Na+ exhibited positive data compared to the synthetic plastic in the laboratory using the EN (166)) standardization. Therefore, it can be concluded that both organic (cellulose and starch) based edible nano-coating bioplastic may be used for drug and capsule coating as biomedical and medical components in the pharmaceutical industries.

**Specification Table**

**Table:** 

Subject area	Biological chemistry, Biochemistry
More specific subject area	Nanocellulose based nanocoating from plant biomass
Type of data	Physicochemical (Table and Figure)
How data were acquired	SEM, pH meter, spectrophotometer, Tensile test was performed by Universal Test Machine, absorption test, burning test, crack test, energy test, chemical test by ASTM and EN standard.
Data format	Row data were collected and analyzed
Experimental factors	Single factor
Experimental features	Three replicates were used in the experiment as Complete Randomized Design (CRD). The sample was selected randomly from the different lots. Standard deviation and standard error was analyzed.
Data source location	Kuala Lumpur, Malaysia and Hail, KSA.
Data accessibility	This is an innovative data, not yet published elsewhere.

**Value of the Data**

1. Data represented are a superlative and an innovative research based work. Data would be a valuable to the researcher those who are doing research on nano-coating production utilizing biomass as plant biomaterial.
2. Data having innovative information on nano-coating for drug and capsule or other definite biomaterials from corn leaf biomass have been explored.
3. Data identify the suitability of nano-cellulose based nano-coating plant biomaterial production using leaf biomass according to the ASTM (American standard for testing and materials) and EN (European Norms) standardization.
4. Data would be highlighted for future studies in the related research community all over the world.

## 1 Data

**Fig. 1:**
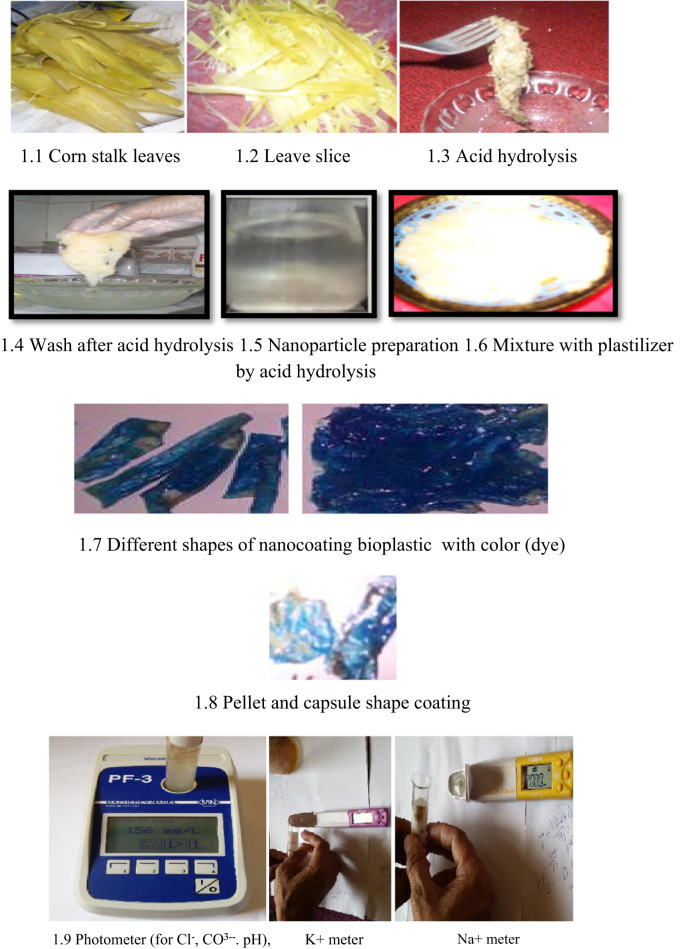
Photographs show the nano-coating bioplastic preparation process and the photometer (for Cl^-^, ${\mathrm{CO}}_{3}^{--}$. pH), K+ meter and Na+ meter (Horiba Scientific, Japan).

**Fig. 2:**
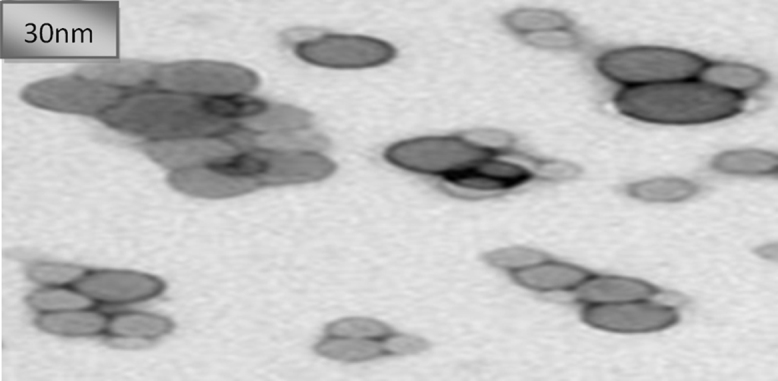
Photo shows the TEM image of nanocellulose from corn stalk leaves fiber.

Fig. 1 shows the nano-coating production procedure from corn leaf biomass. The data exhibit the nanosized particle (30 nm) as nanocellulose detected by Transmission Electron Microscopy (TEM) (Fig. 2). From the data (Table 1), it has been seen that negligible water 0.01% was absorbed by nanocoating bio-plastic.

**Table 1:** Determination of water absorption by ASTM D570.

Materials	Water absorption	ASTM D570
		Water absorption
Nano-coating	0.01%	Water absorption by ASTM is 0–0.16%.
Synthetic plastic	0–0.16%	

In Table 2, data observe the odor represented by burning test was under completely standard of burning test. Data on the color coating time for drying were 1.2 h (Table 3). Table 4 shows the tensile strength 73.0 MPa/kg m^3^ and tensile modulus 1.01 GPa for the nano-coating bioplastic. Moreover, data shown in Table 5 of pH and cellulose and exhibited positive value. The data of chemical element test like K+, ${\mathrm{CO}}_{3}^{--}$, Cl^-^_,_ Na+ were measured and represented positive data compared to the synthetic plastic in the laboratory using the EN (166)) standardization (Table 6).

**Table 2:** Odor test according to the ASTM D3801.

Materials	Odor	Color of flame	Speed of burning	Spark or not
Nano-coating	No odor	Yellow-orange	Slow	Spark
Synthetic plastic				
By ASTM D3801	Low odor	Yellow-orange	Slow	Spark

**Table 3:** Color coating dye was used as the mode of application by ASTM B 117.

Materials	Coating dye test	ASTM B117
	(Drying time)	Maximum 2 h
Nano-coating	1.2 h	Max. 2 h
Synthetic plastic	2 h (max).	
By ASTM B 117

**Table 4:** Determination of tensile test by using ASTM by ASTM D5083.

Materials	Tensile strength (MPa/kg m^3^)	Tensile modulus (GPa)
Nano-coating	73.0	1.01
Synthetic plastic		
By ASTM D5083	70-230 (ASTM)	1.0–3.0 (ASTM)

**Table 5:** pH and cellulose determination from banana waste biomass sample.

Test	pH	Cellulose
Corn samples	7.3 ± 0.01	70.5% ± 0.05
Plastic sample	Alkaline ≥ 7	(It is zero if from gas or oil sample, if from cellulose sample it is 20–80%)

Mean ± SE (standard Error, n = 3)

**Table 6:** Chemical element determination.

Chemical element	Corn sample based biofilm (PPM)	Synthetic plastic By EN (European Standard EN166.)
K+,	9.7 ± 0.5	10
Na+	4.2 ± 0.2	5
Cl^-^	0.55 ± 0.01	2
${\mathrm{CO}}_{3}^{--}$	139.1 ± 1.1	5-440

Mean ± SE (standard Error, n = 3)

## 2 Experimental design, materials and methods

### 2.1 Sample collection and preparation

Five kg corn stalk new leaves were collected from the farmers field, Kuala Lumpur Malaysia and Hail regional area, KSA. Leaves were randomly chosen from both area and removed from corn stalk and washed to clean. Washed leaves were sliced by scissors (Fig. 1). Then it was blended by blender. After blending it was again ground for fine mixing by motor and pestle and put it to the beaker.

### 2.2 pH determination

The pH was determined by using Horiba Scientific pH meter, Japan.

### 2.3 Cellulose determination

#### 2.3.1 Dinitrosalicylic acid (DNS) method for cellulose determination [1]

Cellulose content was determined by 3, 5-dinitrosalicylic acid. A standard curve was drawn by measuring the absorbance of known concentration of cellulose solutions at 575 nm. DNS reagent consisted of 1% dinitrosalicylic acid, 0.2% phenol, 0.05% sodium sulfite and 1% sodium hydroxide. To measure cellulose content, 3 ml of unknown cellulose solution was filled into a test tube, followed by addition of 3 ml of DNS reagent. The test tubes were then heated in boiling water bath for 15 min. One ml of 40% potassium sodium tartrate solution was then added prior to cooling. All test tubes were then cooled under running tap water and its absorbance at 575 nm was measured.

### 2.4 Samples pyrolysis

Blended and ground sample was heated at 150 °C in pressure cooker for 2 hours at 30 psi until the sample was become liquid paste. After heating the liquid fiber samples were cooled down. A 0.8% (w/v) sodium chlorite (NaClO_2_) solution and acetic acid were added to acidify the NaClO_2_ solution until the pH reached 4.5. The fibers were boiled in NaClO_2_ solution for 3 h at 70–80 °C whereby the ratio of fiber to NaClO_2_ solution was set to 1: 30. The bleaching process was repeated for five times until fiber became white and then filtered. After being filtered, the residue was washed for several times with distilled water and dried in air. The bleached cellulose obtained was heated to about 70 to 80 °C in 5% (w/v) sodium sulfite solution for 2 h. The fibers were filtered, washed, and dried in the air. After being dried, the fibers were treated in 17.5% (w/v) sodium hydroxide (NaOH) solution for 2 h. The residue was washed for several times with distilled water.

### 2.5 Nano-particle preparation by acid hydrolysis

Fiber sample was hydrolyzed (100 ml/50 g sample) by hydrochloric acid (H_2_SO_4_ 99% pure) to make it micro to nano size particle for 12 h. The water bath (60 °C) was used during the process of hydrolysis occurred. After 12 h the samples were separated by separation funnel and washed by distilled water five times (Fig. 1).

### 2.6 Nanoparticle measurement

Nano particle size was measured by Transmission electron microscopy (TEM). TEM images were obtained using a JEM-2100 transmission electron microscope operated at 120 kV. For TEM sample preparation, the nanocellulose particles were deposited on a carbon-coated grid by placing a drop of a very dilute cellulose nanofiber suspension on the grid and then allowed to dry in order to evaporate the liquid.

### 2.7 Plasticizer-mixture

Acetic acid 5% (5 ml/100 g sample), 5 ml/100 g (polyvinyl chloride), cellulose (20%) and starch powder 20%, and 20% water were added with the 1000 g of samples. Later 10 ml/100 g PVC (polyvinyl chloride) and glycerin were added with the mixture of samples and waited for 10 min to mix up well. Then the mixture was heated by pyrolysis method as ASTM standard (at 150 °C in the oven for 30 min (at 30 psi pressure) until visual plasticity in the oven for nanocoating bioplastic material. The samples were taken it out from the oven and kept it in room temperature (28 °C) for cooling down for 10 min. It was put in the aluminum foils to make it dry containing as nano-bioplastic. Finally it was oven dried at 80 °C. The nanocoating bioplastic was used for different test for fitness.

### 2.8 Testing fitness

Absorption test as ASTM [2].

For the water absorption test, the specimens were dried in an oven for a specified time and temperature and then placed into the desiccator to cool. Immediately upon cooling the specimens were weighed. The material was then emerged in water at agreed upon conditions, often 23 °C for 24 h. Specimens were removed, patted dry with a lint free cloth, and weighed (Table 1). The diameter of disk was 5 cm and 2 mm thick. Water absorption was calculated.

### 2.9 Odor test

**Fig. 3:**
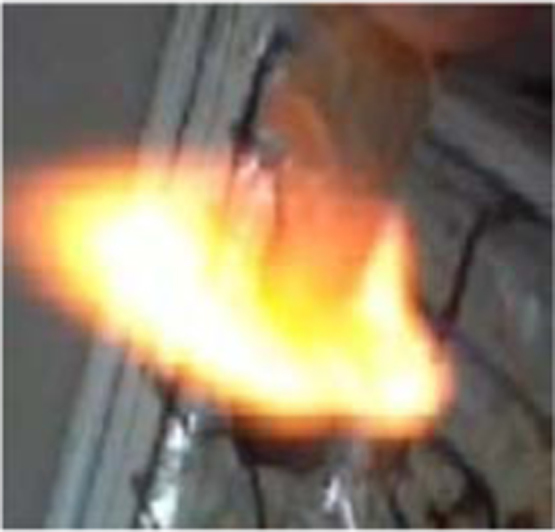
Burning test of nanocoating biomaterial.

It was burnt by using gas burner. Odor, color of flame, speed of burning and spark were observed by visual observation and compared with the synthetic plastic by ASTM D3801 (Fig. 3).

### 2.10 Tensile/tension test

Tensile test was done by Universal Test Machine for bioPlastics as ASTM [3].

### 2.11 Test Procedure

Specimens were placed in the grips of a Universal Test Machine at a specified grip separation and pulled until failure. For ASTM D5083 the test speed was measured by the material specification. The default test speed was 5 mm/min (0.2 in/min), but modulus determination was made at 1 mm/min (0.039 in/min). A strain gauge was used to determine the elongation and tensile modulus. Max Load Capacity was 50 kN/m^2^ depending upon the reinforcement and type.

#### 2.11.1 Sample size

The standard specimen for ASTM [3] has a constant rectangular cross section, 25 mm (1 in.) wide and at least 250 mm (10 mm) long. Thickness can be between 1 mm (0.039 in.) and 14 mm (0.55 in.). Optional tabs can be bonded to the ends of the specimen to prevent gripping damage. Intertek PTL can machine the specimens from larger samples and bond tabs if requested. Tensile Strength (MPa or PSI) was displayed from tensile test.

### 2.12 Color test

Spray coating dye was used as the mode of application. It was attached properly with plastic and dried after 1 h (Fig. 1).

### 2.13 Chemical element test

Chemical element like K+, ${\mathrm{CO}}_{3}^{--}$, Cl^-^_,_ Na+ were tested using different meters. K+ and Na+ were tested by LAQUA twin K+ meter and LAQUA twin Na+ meter (Horiba, Japan) (Fig. 1). CO_3_^-^, and Cl^-^ were tested by Photometer PF-3,version A (Macherey-Nagel, Germany). In the case of all chemical elements positive results exhibited and compared to the synthetic plastic in the laboratory using the EN (166)) standardization [4].

### 2.14 Statistical analysis

Randomized Complete Design (CRD) was employed. The sample was selected randomly from the different lots in the experiment. Standard deviation was calculated from the mean of the replicates and Standard error was analyzed from standard deviation using 3 replicates of the samples where necessary (n = 3) (n = replicate).
